# Diverse Scaffolds Facilitate NLRP3 Clustering and Inflammasome Formation in Response to Perturbations in Cell Homeostasis

**DOI:** 10.1002/bies.70130

**Published:** 2026-03-25

**Authors:** Elvira Boršić‐Mlinarič, Iva Hafner‐Bratkovič

**Affiliations:** ^1^ Department of Synthetic Biology and Immunology National Institute of Chemistry Ljubljana Slovenia; ^2^ Interdisciplinary Doctoral Study of Biomedicine Faculty of Medicine University of Ljubljana Ljubljana Slovenia; ^3^ Institute of Cell Biology Faculty of Medicine University of Ljubljana Ljubljana Slovenia; ^4^ EN‐FIST Centre of Excellence Ljubljana Slovenia

## Abstract

A central component of innate immunity, the NLRP3 inflammasome is a multiprotein complex formed in response to a chemically and morphologically diverse spectrum of stimuli. Despite extensive investigation, no single ligand or signal has emerged to account for this breadth of activation. Here, we review the landscape of NLRP3 activation across subcellular compartments and examine how this process is shaped by a network of interacting partners. Recent studies suggest that NLRP3 responds to cellular perturbations, such as changes in lipid membrane composition, protein localization, or organelle function. We propose that distinct upstream cues converge to generate diverse molecular scaffolds that recruit NLRP3. The NLRP3‐scaffold interactions promote NLRP3 clustering, destabilize its autoinhibited conformation, and drive assembly of the inflammasome. NLRP3 is thus an adaptable sensor, equipped with versatile molecular interactions that allow it to integrate multiple danger signals into inflammasome activation.

## Graphical abstract

NLRP3, a cytosolic innate immune sensor, responds to diverse activators that perturb cellular homeostasis. These disturbances generate molecular scaffolds that, through binding partners such as nucleic acids, lipids, and proteins, recruit and concentrate NLRP3. Scaffold‐mediated NLRP3 clustering promotes NLRP3 inflammasome assembly.

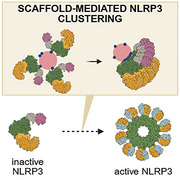

## 1 Introduction

Inflammasomes are cytosolic multiprotein complexes that play critical roles in innate immunity. Among them, the NLRP3 (NLR family protein containing a pyrin domain 3) inflammasome assembles in response to diverse pathogen‐associated molecular patterns (PAMPs) and danger‐associated molecular patterns (DAMPs) and has been shown to be involved in various diseases, including neurodegeneration, metabolic disorders, and cancer [1, 2]. Gain‐of‐function mutations in the *NLRP3* gene cause rare cryopyrin‐associated periodic syndromes (CAPS), characterized by systemic inflammation that are currently treated with anti‐interleukin (IL)‐1‐targeted therapies [3, 4]. Due to its involvement in multiple modern‐day diseases, several NLRP3 inhibitors are currently being evaluated in clinical trials [5].

Upon activation, NLRP3 forms a disc‐like structure [6] and recruits the adaptor ASC (apoptosis‐associated speck‐like protein containing a CARD), enabling autoproteolytic activation of pro‐caspase‐1. Active caspase‐1 cleaves pro‐IL‐1β and pro‐IL‐18 into their active forms and gasdermin D, releasing its N‐terminal domain to oligomerize into pores in the plasma membrane, thereby inducing pyroptosis [7, 8]. NLRP3 consists of an N‐terminal pyrin domain (PYD), a central NACHT domain (present in NAIP, CIITA, HET‐E, and TP1), and a C‐terminal leucine‐rich repeat (LRR) domain. Contrary to the canonical activating ligand‐binding role of the LRR domain in toll‐like receptor (TLR) signaling, the sensing region for different activators has been mapped to the linker‐FISNA (fish‐specific NACHT‐associated) domain [9, 10], positioned next to the PYD domain that enables homotypic interactions with ASC. The LRR domains do not directly contribute to active oligomer formation [6] and are not necessary for NLRP3 inflammasome activation [11], yet they might potentiate it [12]. The LRR domain regulates NLRP3 at several levels. It has been shown to stabilize NLRP3 protein levels [11, 13, 14] and dephosphorylation of S803 facilitates NEK7 (NIMA‐related kinase 7) binding [14]. Furthermore, LRR domains drive assembly of cage‐like inactive NLRP3 oligomers, which prevent premature engagement with ASC [15, 16, 17, 18]. Upon activation, several structural transitions are thus needed to facilitate the disassembly or remodeling of inhibitory cages into a decameric disc‐like structure, where the major interaction surface is through FISNA‐NACHT domains [6]. While these seminal structural studies illustrate the complexity of NLRP3‐NLRP3 interactions in inactive and active oligomers, the molecular mechanisms that govern the trigger‐dependent transition to the active NLRP3 form and inflammasome are not fully understood. NLRP3's activity is tightly controlled at the transcriptional, post‐transcriptional, and post‐translational levels (recently reviewed in [19, 20, 21]), yet how these regulatory layers converge to relieve autoinhibition and promote inflammasome assembly remains to be fully elucidated. We recently identified the importance of NLRP3 association with different molecular scaffolds, as this binding loosens inhibitory interactions and promotes clustering of NLRP3 molecules, leading to inflammasome formation [22].

The enigmatic feature of NLRP3 remains its ability to respond to a variety of chemically and structurally diverse activators. Accumulating evidence presents a number of molecular mechanisms activating NLRP3, suggesting that NLRP3 may not be activated by direct recognition of a single molecular species, but rather through sensing versatile cellular disturbances [22], ranging from potassium efflux [23, 24, 25] to organelle dysfunction [26], supporting the concept that NLRP3 responds to homeostasis‐altering molecular processes [27, 28].

In this review, we summarize and discuss recent advances that provide insight into how NLRP3 can respond to a broad range of signals. We review studies demonstrating that NLRP3 can be activated at various subcellular locations. Further, we describe multiple NLRP3 binding partners and molecular scaffolds that mediate NLRP3 binding through different domains to drive inflammasome assembly. We propose that this well‐coordinated plasticity enables NLRP3 to respond to various perturbations in cell homeostasis.

## 2 Scaffold‐Mediated NLRP3 Clustering Occurs at Diverse Subcellular Locations

Canonical NLRP3 inflammasome activation is a two‐step process. The first signal, priming, is often provided by TLR agonists such as lipopolysaccharide (LPS), which upregulate transcription of NLRP3 and pro‐IL‐1β and initiate post‐translational modifications of NLRP3 [29]. The second, activation signal, is triggered by a panel of PAMPs and DAMPs, such as pathogens, microbial toxins, and ATP [30, 31, 32], environmental particles, and endogenous crystals [33, 34, 35, 36], aggregated proteins [37, 38] and intracellular danger signals [39, 40]. It was proposed that several instigators induce potassium efflux [23, 24, 25]; however, imidazoquinolines activate the inflammasome independently [41], pointing toward alternative mechanisms.

Recently, the importance of NLRP3's subcellular localization has received considerable attention (reviewed in [42, 43]). During inflammasome activation, NLRP3 was observed in the vicinity of distinct subcellular compartments, particularly the endoplasmic reticulum (ER), Golgi apparatus (GA), mitochondria, and endosomes. For example, the SREBP2/SCAP complex is responsible for transferring NLRP3 from the ER to the GA [44]. Herpes simplex virus type 1 (HSV‐1) infection induces recruitment of NLRP3 to the ER through stimulator of interferon genes (STING), leading to NLRP3's deubiquitination and inflammasome activation [45]. Other studies show that in the resting state, NLRP3 predominantly localizes to the ER, and upon activation, it was observed to translocate to mitochondria‐associated membranes (MAMs) [39, 46]. On the other hand, inflammasome formation requires NLRP3 to be released from MAMs through phosphorylation of NLRP3 at serine 295 by protein kinase D (PKD) [47]. Arumugam and colleagues reported that NLRP3 stimuli activate glycogen synthase kinase 3β (GSK3β), which drives NLRP3 recruitment first to mitochondria and then to the GA. Through activation of phosphatidylinositol 4‐kinase 2A, GSK3β increases phosphatidylinositol‐4‐phosphate (PI4P) at the trans‐Golgi network (TGN) [48], a lipid shown by Chen and Chen to be critical for NLRP3 recruitment and activation at the disassembled TGN [49]. Notably, NLRP3 contains a basic cluster (residues 127–146) that directly interacts with PI4P [49], and dispersion of the TGN could change the curvature of TGN vesicles, thereby inducing NLRP3 oligomerization [49]. Recent studies report that these vesicles originate from endosomes [50, 51], suggesting that endosomal PI4P pools may also contribute to NLRP3 activation. Furthermore, NLRP3 can be transported to the microtubule‐organizing center (MTOC) [52], a process facilitated by microtubule affinity‐regulating kinase 4 (MARK4) and HDAC6 [53, 54], where it can directly interact with NEK7 and form the inflammasome [15, 55]. Recently, Mateo‐Tortola and colleagues identified two parallel NLRP3 activation pathways in human cells, one MTOC‐dependent and the other MTOC‐independent, both activated in response to nigericin [56]. Interestingly, HDAC6 not only facilitates NLRP3 positioning at the MTOC but also enables interaction of NLRP3 with Lamtor1 on lysosomes [57]. Lysosomes are important for NLRP3 activation, as their destabilization in response to particulate triggers leads to inflammasome activation [33]. Lysosomal positioning at the cell periphery increased NLRP3 inflammasome activation in response to nigericin, which correlated with changed subcellular location of the formed inflammasome [58].

**FIGURE 1:**
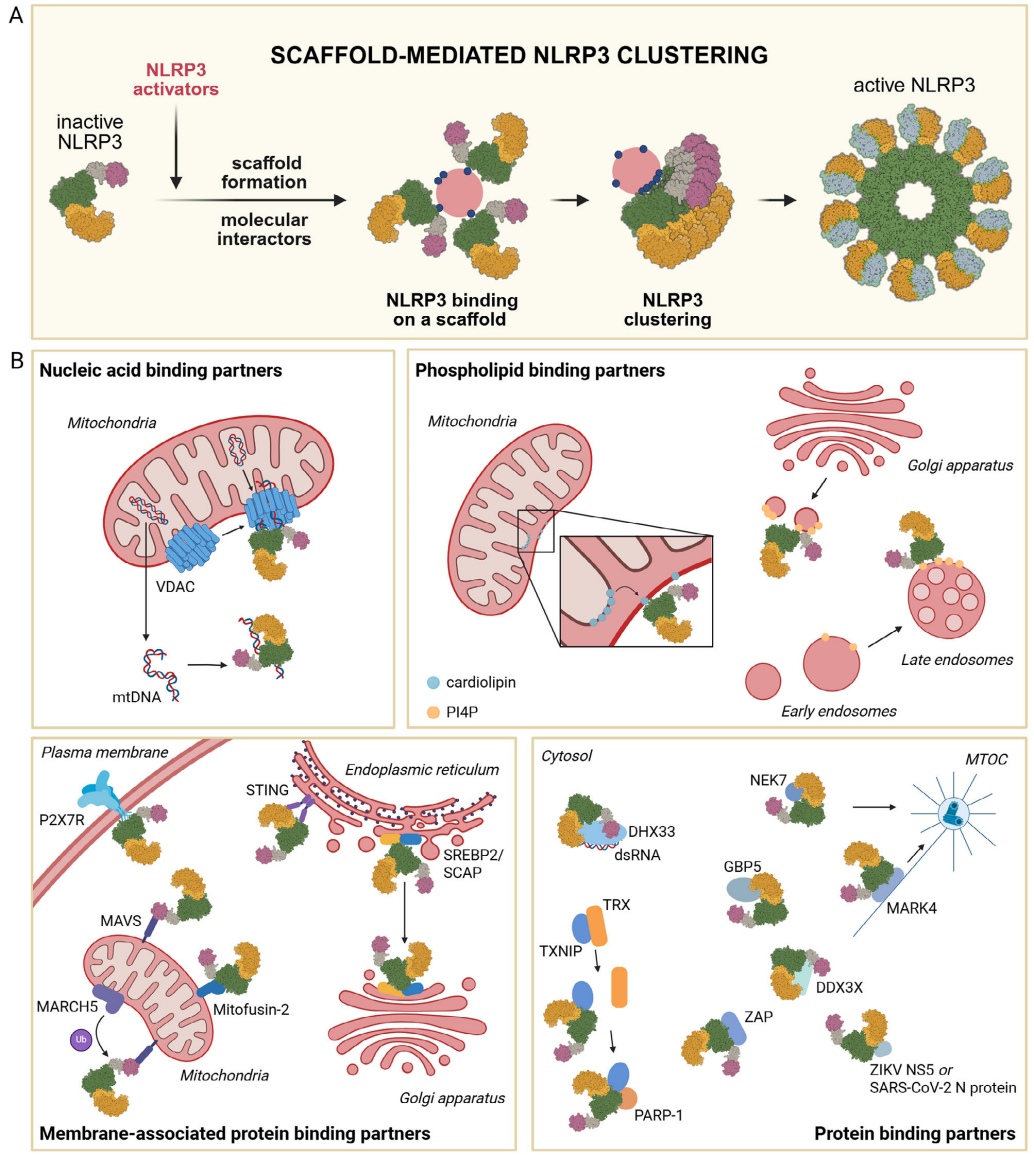
Molecular scaffolds drive NLRP3 inflammasome activation. (A) NLRP3 activators induce the formation of diverse molecular scaffolds that recruit NLRP3, either directly or through binding partners (B), and enable its clustering, stabilizing the active conformation and facilitating the assembly of the NLRP3 inflammasome. Models of NLRP3 were generated based on 7PZC (inactive NLRP3) [16] and 8EJ4 (NLRP3 inflammasome) [6]. NLRP3 domains are depicted in purple (PYD), grey (linker), green (NACHT), light orange (transition LRR) and dark orange (LRR). (B) Distinct binding partners promote NLRP3 inflammasome activation. Created in Biorender.

While several subcellular locations, ranging from ER, MAMs/mitochondria, GA, endosomes, lysosomes, or MTOC, seem to promote NLRP3 inflammasome assembly, recent studies suggest that an exclusive location may not be strictly required for inflammasome activation. Extensive proteomic analysis has revealed that NLRP3 agonists induce large perturbations in the cell [59]. By directing NLRP3 to distinct subcellular locations, we demonstrated that NLRP3 can be activated by both potassium efflux‐dependent and independent triggers, regardless of its predominant position within the cell [22]. However, binding to molecular scaffolds, either membrane‐ or protein‐based, is important, as it enables the clustering of NLRP3 molecules (Figure 1A). Using a panel of NLRP3 inhibitors with well‐defined modes of action, we proposed that clustering enables the loosening of the inactive conformation, but not at the level of disrupting LRR interactions as has been shown for NEK7 [6, 15, 16]. Interestingly, in vitro, the active NLRP3 disc could only be formed upon the addition of ASC molecules, which indicates that scaffold associations are necessary not only in the context of the cell but also at the molecular level in isolated in vitro conditions [6].

Cellular stress likely activates NLRP3 indirectly, that is, through changes in lipid composition, membrane curvature, or the abundance and localization of NLRP3 binding partners. These local changes in the cytoplasm create interfaces that can promote NLRP3 association and subsequent inflammasome assembly. In the following sections, we will examine NLRP3 binding partners and associated scaffolds in more detail, emphasizing the versatility of interactions that support inflammasome assembly.

### 2.1 Nucleic Acids Mediate NLRP3 Inflammasome Assembly

Nucleic acids have been implicated as activators of the NLRP3 inflammasome [60, 61, 62, 63], but direct binding has only been demonstrated for mitochondrial DNA (mtDNA) [64, 65, 66, 67] (Figure 1B). mtDNA is released during cellular stress and recognized by different pattern recognition receptors (PRR), such as TLR9, cGAS‐STING, AIM2, and NLRP3, with several studies showing a preference of NLRP3 for oxidized DNA (ox‐mtDNA) [64, 66, 67]. Interestingly, production of ox‐mtDNA was significantly increased in bone marrow‐derived macrophages (BMDMs) when treated with LPS and ATP compared to LPS alone [64]. Interaction between NLRP3 and mtDNA has been observed using purified protein [67] and across different cell types, such as Kupffer cells (liver resident macrophages) [65], BMDMs [66], and HEK293T cells [64], under varying stimulating conditions, indicating that mtDNA is a broadly involved NLRP3 binding partner. Notably, two CAPS mutant variants, L355P and L266F, displayed no characteristic binding of ox‐mtDNA in the electrophoretic mobility shift assay compared to the wild‐type variant [67], consistent with constitutively active NLRP3 variants bypassing the need for a scaffold to facilitate oligomerization [22].

Common inflammasome instigators induce hexokinase dissociation from the voltage‐dependent anion channel (VDAC) on the mitochondrial membrane, triggering its oligomerization and macromolecular pore formation that can pass mtDNA fragments [68, 69]. mtDNA likely facilitates inflammasome activation through direct binding to NLRP3 in the vicinity of VDAC pores (Figure 1B), as depletion of mtDNA with ethidium bromide markedly reduced IL‐1β release despite intact VDAC oligomerization [68].

In conclusion, multiple studies indicate that mtDNA serves as a nucleic acid binding partner, with evidence of direct engagement with NLRP3 to form the inflammasome.

### 2.2 Phospholipids Facilitate NLRP3 Recruitment to Membranes of Various Organelles and Vesicles

Several layers of evidence indicate that membrane association is important for NLRP3 inflammasome activation. In 2013, Iyer and colleagues identified a direct interaction between NLRP3 and cardiolipin [40], a phospholipid normally localized in the inner mitochondrial membrane that becomes exposed on the outer membrane during mitochondrial damage (Figure 1B). Subsequent studies confirmed this by demonstrating that cardiolipin becomes accessible during priming, resulting in NLRP3 association with mitochondria [70] and that the inactive dodecameric NLRP3 cage strongly interacts with cardiolipin [17].

Chen and Chen identified PI4P as a key lipid that binds NLRP3. Based on colocalization with GA markers, they proposed that NLRP3 is recruited to PI4P‐enriched dispersed TGN vesicles [49] (Figure 1B). Targeted mutagenesis of the polybasic region (residues 127–130 in mouse NLRP3) demonstrated that charge interactions are critical for binding and disruption of this region abolished PI4P engagement [49]. This interaction appears to involve inactive NLRP3, as inactive NLRP3 cages associate with the TGN [15], and NLRP3 rapidly localizes to PI4P‐rich membranes within 30 min of LPS priming [71]. Interestingly, when NLRP3 was constitutively bound to different membranes via organelle‐specific tags, it could still form the inflammasome regardless of the presence of the polybasic segment [22], suggesting that membrane localization, rather than specific PI4P binding, may be the critical functional requirement. Recently, two studies demonstrated that NLRP3 is recruited to PI4P‐rich vesicles of endosomal origin [50, 51] (Figure 1B), which was further supported by a proteomic study [59]. NLRP3 can also associate with PI(3,5)P_2_ on acidic vesicles [72].

The importance of PI4P binding for NLRP3 activation appears to differ between species. Mutating the polybasic motif KMKK in human NLRP3 only mildly impaired activation by nigericin [9], in contrast to findings in mouse NLRP3, where equivalent mutations were more disruptive [22, 49]. Notably, NLRP3 contains a second polybasic stretch within the FISNA domain (135‐145 in human NLRP3), which could, in principle, mediate lipid interactions. In a recent preprint, Gravastrand and colleagues demonstrated that human NLRP3 is translocated to PI4P‐positive membranes via Rab11‐FIP2, which binds to the KMKK motif, suggesting a possible compensatory mechanism [73].

As outlined in the preceding sections, in the cellular context, NLRP3 was shown to bind cardiolipin, PI4P, and PI(3,5)P_2_. However, in vitro studies reveal that NLRP3 has an even broader spectrum of lipid interactions. Full‐length murine NLRP3 and the isolated polybasic region of NLRP3 also bound other phosphoinositides and phosphatidic acid [15, 49], whereas human NLRP3 interacted primarily with phosphatidic acid and PI(3,4,5)P_3_ [56]. Although in vitro lipid‐binding assays warrant confirmation in living cells, it is known that phosphoinositides are differentially present in the membranes of different organelles [74], which further advocates for the possibility of NLRP3 inflammasome assembly at different organellar membranes [22].

Adding another layer of membrane interactions, recent studies uncovered that NLRP3 undergoes a reversible posttranslational modification, palmitoylation, at several cysteines, which promotes inflammasome activation and binding of NLRP3 to the membranes, especially during priming stages [75, 76, 77, 78].

While phospholipids are tightly regulated in both abundance and distribution, NLRP3 is able to interact with multiple phospholipid species, enabling its association with diverse membranes. Although membrane association alone is not sufficient for inflammasome activation [22, 49, 71], membranes of different organelles can serve as scaffolds that allow NLRP3 to respond to changes in membrane environments, such as curvature or local lipid composition.

### 2.3 Protein Partners Coordinate NLRP3 Membrane Association

In addition to lipids, transmembrane or membrane‐anchored proteins spatially coordinate NLRP3 recruitment and activation (Figure 1B). Such protein platforms can have dual roles, either by bringing NLRP3 proximally to membranes or by nucleating NLRP3 into inflammasome formation.

Upon viral infection, mitochondrial antiviral signaling protein (MAVS) induces type I IFN production and NF‐κB activation [79, 80, 81], but also mediates NLRP3's recruitment to mitochondria and promotes its oligomerization [82, 83]. Notably, a MAVS variant lacking the transmembrane region required for mitochondrial localization was unable to scaffold NLRP3 oligomerization [82], underscoring the importance of membrane anchoring in this process. Sendai virus infection itself induced NLRP3 oligomer formation, but less efficiently than when MAVS was overexpressed, and this effect was markedly reduced by MAVS knockdown [82]. While NLRP3 can oligomerize in the absence of MAVS, MAVS acts as a potent scaffold that strongly enhances and stabilizes oligomer formation during infection.

MARCH5, an E3 ubiquitin ligase located at the outer mitochondrial membrane, was shown to mediate K27‐linked polyubiquitination on the K324 and K430 residues of NLRP3. This modification promoted NLRP3 oligomerization, as NLRP3 puncta were not present in *March5* conditional knockout BMDMs stimulated with LPS and nigericin or ATP [84]. Interestingly, MAVS mediated mitochondrial localization and interaction with MARCH5 [84], suggesting a combinatorial mechanism of scaffolding in which MAVS positions NLRP3 at mitochondria and MARCH5 modifies it to promote oligomerization. Similarly, mitofusin‐2, a key regulator of mitochondrial fusion, facilitates NLRP3 recruitment to mitochondria via direct binding during RNA virus infection [85].

STING is another important adaptor in innate immunity, mediating the inflammatory response to cytosolic DNA. HSV‐1 infection and transfection of HSV120, a synthetic DNA fragment from the HSV‐1 genome, induced STING‐dependent NLRP3 inflammasome activation in multiple cell systems through recruiting NLRP3 to the ER and serving as a nucleation site for inflammasome assembly. Direct interaction between STING and NLRP3 resulted in reduced K48‐ and K63‐linked polyubiquitination, thereby further promoting NLRP3 activation [45]. Beyond infection, recent evidence from cerebral ischemia models showed that STING directly interacts with NLRP3 in vivo and in cultured microglia, driving inflammasome activation and pyroptosis, thereby exacerbating neuroinflammation and brain injury [86].

Besides its canonical function in regulating cholesterol homeostasis, genetic disruption of the SCAP–SREBP2 complex impaired NLRP3 activation. Mechanistically, SCAP–SREBP2 complex promoted enrichment of NLRP3 at the GA through translocation from the ER, which enabled inflammasome activation adjacent to mitochondria [44].

Activation of purinergic P2 × 7 receptor (P2 × 7R) by extracellular ATP mediates NLRP3 inflammasome activation [87]. Beyond that, activation of P2 × 7R in microglia sequesters NLRP3 in subplasmalemmal regions through direct interaction with the receptor [88]. Consistent with this spatial confinement of NLRP3 at the membrane through binding partners, we demonstrated that directing NLRP3 to different membranes using organelle‐specific protein targeting sequences allows both potassium‐dependent and independent triggers to induce inflammasome assembly regardless of the membrane type [22]. Not only did these tags spatially restrict NLRP3, but one of them, a giantin‐derived membrane‐targeting sequence, also induced NLRP3 clustering on the membrane, leading to inflammasome activation in the absence of triggers, pointing toward the role of NLRP3 binding partners [22].

These studies demonstrate that membrane‐associated proteins across different organelles enhance NLRP3 activation through diverse mechanisms, either by promoting oligomerization or by facilitating post‐translational modifications and trafficking of NLRP3 into proximity of additional binding partners, such as mtDNA, that ultimately drive inflammasome assembly.

### 2.4 Non‐Membrane‐Associated Proteins Promote Inflammasome Activation

Beyond membranes, proteins can provide organizational platforms for NLRP3 inflammasome formation (Figure 1B). The most extensively studied partner promoting NLRP3 inflammasome assembly is NEK7. Typically a centrosome protein, NEK7 was shown to interact with NLRP3 and stabilize the open conformation required for oligomerization in response to potassium efflux [6, 89, 90, 91, 92]. Yet, NEK7 may not always be required, since under certain conditions, NLRP3 can be activated independently [71, 93].

The interferon‐inducible GTPase guanylate‐binding protein 5 (GBP5) was identified as an inducer of NLRP3 oligomerization, as GTPase‐dependent tetramerization greatly enhanced ASC speck formation, and inflammasome activation was reduced in Gbp5^–/–^ mice [94]. Similarly, the cytoskeletal protein MARK4 binds NLRP3 and facilitates its transport to the MTOC, where the local high concentration of NLRP3 enables inflammasome assembly [53]. Given that a lot of reports suggest that NLRP3 assembles at the MTOC [15, 54, 56], this organelle itself could serve as a sufficient protein scaffold to initiate inflammasome assembly, as NLRP3 fused to a centrosomal targeting sequence activated the inflammasome in the absence of canonical instigators [22]. An MTOC‐independent pathway has also been proposed [56], exemplified by synthetic TDP43‐based liquid condensates, where phase separation provides a dynamic protein scaffold for inflammasome assembly [22].

Other proteins were reported to facilitate inflammasome formation. RNA helicase DHX33 interacts with NLRP3 upon sensing double‐stranded RNA [95]. Similarly, DDX3X binds NLRP3 in the primed and activated state, promoting NLRP3 oligomerization [96]. Under oxidative stress, thioredoxin‐interacting protein (TXNIP) dissociates from thioredoxin and directly binds NLRP3 [97, 98], and this complex formation can be further mediated by poly(ADP‒ribose) polymerase‐1 [99]. Another member of the PARP family, zinc‐finger antiviral protein (ZAP), was recently found to enhance NLRP3 oligomerization through direct interaction in response to a wide range of stimuli [100]. NLRP3 can also associate with other NLR family members such as NLRC4 [101] and NLRP11 [102]. Not only endogenous proteins, but also viral proteins, can act as scaffolds. For instance, the Zika virus (ZIKV) NS5 protein directly mediated inflammasome assembly and IL‐1β secretion during infection [103], as well as SARS‐CoV‐2 N protein [104].

Collectively, cytosolic proteins can provide diverse and adaptable platforms for NLRP3 inflammasome assembly in response to a heterogeneous set of cellular disturbances.

### 2.5 Different NLRP3 Domains Facilitate Binding to Partner Molecules

It is intriguing how interactions between NLRP3 and such a wide panel of molecules can all lead to inflammasome activation. The majority of known binding partners shown to activate the NLRP3 inflammasome associate with either the NACHT or LRR domain (Table 1). While binding to the LRR domains could disrupt the inactive NLRP3 cage, PYD domains need to be exposed for ASC recruitment during inflammasome assembly. Notably, GBP5 via its GTPase domain was demonstrated to interact with the PYD domain of both human and mouse NLRP3, and inflammasome activation was impaired in GBP5‐deficient BMMs [94]. Similarly, MAVS and MARK4 have also been shown to interact with the N‐terminal PYD [83] or the PYD–NACHT region of NLRP3 [53], respectively. MARK4 presumably binds within the PYD domain, as the NLRP3 V52G point mutation significantly impaired the interaction [53].

**TABLE 1:** Binding partners of NLRP3 that promote inflammasome activation.

Partner	NLRP3 region	Domain	Method	References
GBP5	hNLRP3^1‐114^	PYD	GST pull‐down	[94]
MAVS	hNLRP3^2‐7^	PYD	Immunoprecipitation	[83]
MAVS	hNLRP3	PYD NACHT	Immunoprecipitation	[82]
MARK4	hNLRP3^1‐219^ hNLRP3^V52G^ showed reduced binding	PYD, PYD linker	GST pull‐down, genetic mutation	[53]
PI4P	mNLRP3^127–130^	PYD linker	Lipid blot assay	[49]
PI3P	mNLRP3^127–130^	PYD linker	Lipid blot assay	[49]
PI(3,5)P2	mNLRP3^127–130^	PYD linker	Lipid blot assay	[49]
PI(4,5)P2	mNLRP3^127–130^	PYD linker	Lipid blot assay	[49]
PA	mNLRP3^127–130^	PYD linker	Lipid blot assay	[49]
PA	hNLRP3^95‐134^	PYD linker	Lipid blot assay	[56]
PI(3,4,5)P_3_	hNLRP3^95‐134^	PYD linker	Lipid blot assay	[56]
FIP2	hNLRP3^131–134^	PYD linker	Flag tag pull‐down, genetic mutation	[73]
PRRSV‐2 nsp2	hNLRP3^94‐534^	PYD, NACHT	Immunoprecipitation	[105]
DHX33	hNLRP3^220–574^	NACHT	Immunoprecipitation	[95]
SREBP/SCAP		NACHT	Immunoprecipitation	[44]
MARCH5	hNLRP3	NACHT	Immunoprecipitation	[84]
UCH‐L1	hNLRP3^140‐700^	NACHT	Immunoprecipitation	[106]
Cardiolipin	hNLRP3^535‐719^	HD2, NACHT‐LRR linker region	Binding to cardiolipin‐coated beads	[40]
ZIKV NS5	hNLRP3^121‐600;^ hNLRP3^601‐1036^	NACHT, LRR	Immunoprecipitation	[103]
ZIKV NS5	hNLRP3^601‐1036^	LRR	GST pull‐down, yeast two‐hybrid	[103]
TRIM50	hNLRP3^219‐741^	NACHT linker	Immunoprecipitation	[107]
NEK7	hNLRP3^220–536^ hNLRP3^742‐991^	NOD LRR	Immunoprecipitation	[89]
NEK7	hNLRP3	NACHT (HD2) LRR	Structure	[91]
STING	hNLRP3^121‐1036^	NACHT, LRR	Immunoprecipitation	[45]
STAT3	hNLRP3	PYD, LRR	Immunoprecipitation, proximity ligation assay	[108]
TXNIP	hNLRP3	NACHT, LRR	Yeast two‐hybrid, immunoprecipitation	[97]

Abbreviation: GST, Glutathione S‐transferase.

The linker‐FISNA region, located between the PYD and NACHT domains, was shown to be important for the binding of several negatively charged phospholipids due to its high abundance of basic residues [49, 56]. FIP2 also binds to this polybasic region, enabling NLRP3 recruitment to PIP4‐positive endosomes [73].

The central NACHT domain serves as the primary hub for molecular interactions. Binding to the NACHT domain can directly influence conformational transitions within NLRP3 and stabilize its active conformation, as evidenced by CAPS mutations, which predominantly originate in this region [4]. Several interaction partners, including DHX33 [95], SCAP–SREBP2 [44], MARCH5 [84], UCH‐L1 [106], and cardiolipin [40] target the NACHT domain to promote inflammasome assembly.

The LRR domain is crucial for inactive NLRP3 self‐association [15, 16, 17, 18] and contributes to additional binding surfaces, often acting in concert with the NACHT domain. NEK7 [89, 91], TXNIP [97], STING [45], and the viral protein ZIKV NS5 [103] have all been reported to interact with both domains. Such interactions may disrupt LRR‐LRR interactions and help the caged oligomer to fall apart [15, 16].

Together, different domains of NLRP3 facilitate association with partners and recruitment of NLRP3 molecules to scaffolds of different origins. This modularity likely enables broad responsiveness to chemically and structurally distinct stimuli, allowing NLRP3 inflammasome activation.

### 2.6 Concluding Thoughts

Understanding the NLRP3 inflammasome activation mechanism has been the focus of intense investigation for the past two decades, driven by its central role in host defense and its involvement in widespread diseases. Despite extensive efforts to identify a unifying ligand or signal for NLRP3 activation common to a broad range of known instigators, to date, no such ligand or signal has been reported. As reviewed here, several subcellular locations, binding partners, and mechanisms of activation have been proposed, and the observed variation is likely due to the availability of binding partners in different conditions, cell types, and species‐specific contexts (recently reviewed in [109]).

It is attractive to postulate that NLRP3 is an adaptor molecule enabling the signaling response rather than a receptor or sensor, given the number of diverse interaction partners that engage distinct NLRP3 domains. However, the defining feature of a sensor is not necessarily direct ligand binding, but the ability to detect and respond to perturbations in cellular homeostasis triggered by diverse NLRP3 activators. We propose that such perturbations enable the formation of different molecular scaffolds able to bind NLRP3 and induce its clustering, ultimately leading to inflammasome activation. A question remains as to what constitutes a functional scaffold for NLRP3 activation. As several studies report inflammasome assembly on diverse organelles and vesicular structures, such scaffolds might easily concentrate NLRP3 in a confined subcellular volume. However, increased local abundance is not sufficient [22] as the scaffold needs to license conformational transition into the active form, exposing PYD domains for ASC engagement. For the recruitment of ASC, the minimal requirement might be PYD trimerization [110], although higher‐order PYD assemblies are more stable, as suggested by structural observations of heterogeneous particles during active disc formation [6, 111]. Unlike NLRC4‐containing inflammasomes, NLRP3 inflammasome activation was not shown to be amplified at the level of NLRP3, which increases the threshold for NLRP3 inflammasome activation [11]. Perhaps thus NLRP3 depends on scaffolds to concentrate and form a PYD oligomer that enables ASC recruitment.

While some NLRP3 binding partners function as scaffolds, others orchestrate the intracellular transport, implement post‐translational modifications that enable scaffold binding, or stabilize intermediate or active conformations. Of note, some NLRP3 binding partners (reviewed elsewhere, see [112]) exert negative regulation, underscoring that association per se does not necessarily equal activation. Dissecting which interactions or scaffolds induce clustering or licensing in specific contexts remains an open challenge.

NLRP3 is able to integrate a wide variety of molecular inputs, such as cellular stress, microbial infections, and metabolic disturbances. This review provides a unifying framework that integrates seemingly contradictory findings into a coherent model of scaffold‐based NLRP3 activation, positioning NLRP3 as an important sensor of cellular well‐being.

## Author Contributions

**Elvira Boršić‐Mlinarič**: conceptualization, visualization, writing – original draft. **Iva Hafner‐Bratkovič**: writing – review and editing. Both authors reviewed and approved the final version of the manuscript.

## Declaration of Generative AI and AI‐Assisted Technologies in the Writing Process

During the preparation of this review, the authors used ChatGPT, InstaText and Grammarly to assist in editing for language (grammar) use, fluency, and clarity. This tool has not been used in a way to introduce AI‐generated ideas. The authors reviewed and edited the content as needed and take full responsibility for the content of the publication.

## Funding

I.H.B. is supported by Slovenian Research and Innovation Agency (ARIS) grants N3‐0358, J3‐60056, P4‐0176 and European Union EIC PathFinderOpen project Scalpel (grant agreement no. 101185509). E.B.M. is supported by ARIS young researcher PhD grant. I.H.B. is grateful to the European Federation of Immunological Societies for the Eastern Star Award 2022.

## Conflicts of Interest

The authors declare no conflicts of interest.

## Data Availability

Data sharing not applicable to this article as no datasets were generated or analyzed during the current study.
